# Effects of sub-inhibitory concentrations of nafcillin, vancomycin, ciprofloxacin, and rifampin on biofilm formation of clinical methicillin-resistant *Staphylococcus aureus*

**DOI:** 10.1128/spectrum.03412-23

**Published:** 2024-04-23

**Authors:** Ki-Ho Park, Dokyoung Kim, Minji Jung, Dong Youn Kim, Yu-Mi Lee, Mi Suk Lee, Kyung-Wook Hong, In-Gyu Bae, Sun In Hong, Oh-Hyun Cho

**Affiliations:** 1 Division of Infectious Diseases, Department of Internal Medicine, Kyung Hee University College of Medicine, Kyung Hee University Hospital, Seoul, South Korea; 2 Department of Anatomy and Neurobiology, College of Medicine, Kyung Hee University, Seoul, South Korea; 3 Department of Biomedical Science, Graduate School, Kyung Hee University, Seoul, South Korea; 4 Center for Converging Humanities, Kyung Hee University, Seoul, South Korea; 5 Medical Research Center for Bioreaction to Reactive Oxygen Species and Biomedical Science Institute, Graduate School, Kyung Hee University, Seoul, South Korea; 6 Division of Infectious Diseases, Department of Internal Medicine, Kyung Hee University Hospital, Seoul, South Korea; 7 Department of Internal Medicine, Gyeongsang National University Hospital, Gyeongsang National University College of Medicine, Jinju, South Korea; 8 Division of Infectious Diseases, Department of Internal Medicine, Soonchunhyang University Cheonan Hospital, Soonchunhyang University College of Medicine, Cheonan, South Korea; Innovations Therapeutiques et Resistances (INTHERES), Toulouse, France

**Keywords:** *Staphylococcus aureus*, biofilm, nafcilllin, vancomycin, ciprofloxacin, rifampin

## Abstract

Methicillin-resistant *Staphylococcus aureus* (MRSA) infections are often difficult to treat because of their biofilm-forming ability and antimicrobial resistance. We investigated the effects of sub-minimal inhibitory concentrations (MICs) of antibiotics on MRSA biofilm formation. Clinical MRSA isolates were grown with sub-MICs (1/256–1/2 × MICs) of nafcillin, vancomycin, ciprofloxacin, and rifampin. The biofilm biomass was measured using crystal violet staining. Of the 107 MRSA isolates tested, 63 (58.9%) belonged to sequence type 5 (ST5), and 44 (41.1%) belonged to ST72. The MIC_50_/MIC_90_ values of nafcillin, vancomycin, ciprofloxacin, and rifampin were 256/512, 1/2, 64/512, and 0.008/0.03 mg/L, respectively. The sub-MICs of nafcillin, vancomycin, ciprofloxacin, and rifampin promoted biofilm formation in 75 (70.1%), 49 (45.8%), 89 (83.2%), and 89 (83.2%) isolates, respectively. At sub-MICs of nafcillin, the factors associated with strong biofilm induction were the ST5 strain (*P* = 0.001) and *agr* dysfunction (*P* = 0.005). For the sub-MICs of ciprofloxacin, the associated factors were the ST5 strain (*P* = 0.002), staphylococcal protein A type t002 strain (*P* < 0.001), and ciprofloxacin resistance (*P* < 0.001). Among the sub-MICs of rifampin, only ST5 was associated with strong biofilm induction (*P* = 0.006). Because the sub-MICs of rifampin were much lower than clinically relevant concentrations, we further tested the capability of biofilm induction in 0.03-32 mg/L of rifampin. At these concentrations, rifampin-induced biofilm formation was rare in rifampin-susceptible MRSA [1.0% (1 of 100)] but common in rifampin-resistant MRSA [71.4% (5 of 7), *P* < 0.001]. Induction of biofilm biomass at sub-MICs of antibiotics is common in clinical MRSA isolates and is differentially affected by the MRSA strain and antibiotic class.

**IMPORTANCE:**

Bacteria can be exposed to sub-MICs of antibiotics at the beginning and end of a dosing regimen, between doses, or during low-dose therapies. Growing evidence suggests that sub-MICs of antimicrobials can stimulate MRSA biofilm formation and alter the composition of the biofilm matrix. Pevious studies have found that sub-MICs of oxacillin, methicillin, and amoxicillin promote biofilm formation in some community-acquired MRSA (CA-MRSA). We evaluated biofilm induction by sub-MICs of four different classes of antibiotics in 44 CA-MRSA and 63 healthcare-associated MRSA (HA-MRSA) strains. Our study indicated that sub-MICs of nafcillin, vancomycin, ciprofloxacin, and rifampin frequently promote biofilm induction in clinical MRSA isolates. Strong biofilm induction in sub-MICs of nafcillin, ciprofloxacin, and rifampin was more frequent in HA-MRSA than in CA-MRSA. Antibiotic-induced biofilm formation depends on the antibiotic class, MRSA strain, and antibiotic resistance. Our results emphasize the importance of maintaining effective bactericidal concentrations of antibiotics to treat biofilm-related infections.

## INTRODUCTION

Methicillin-resistant *Staphylococcus aureus* (MRSA) is a major cause of hospital-acquired infections. MRSA causes several biofilm-related infections, including infections of central venous catheters, prosthetic joints, heart valves, and postoperative wounds. Biofilms formed by MRSA may resist antibiotic treatment and survive in hostile environments through impaired penetration and evasion of the host immune response. Therefore, MRSA biofilms play a critical role in healthcare-associated infections and infection control (1).

Bacteria can be exposed to sub-minimal inhibitory concentrations (MICs) of antibiotics at the beginning and end of a dosing regimen, between doses, or during low-dose therapies. Growing evidence suggests that the sub-MICs of antimicrobials can stimulate MRSA biofilm formation and change the biofilm matrix composition. Previous studies have found that the sub-MICs of oxacillin, methicillin, and amoxicillin promote biofilm formation in a few types of community-acquired MRSA, such as USA300 (2–4), USA400 (4), and USA500 (4). However, the effects of the sub-MICs of antibiotics on biofilm induction have not been well studied in clinical MRSA isolates, especially in healthcare-associated MRSA strains. Biofilm-related infections involving medical devices occur more frequently in hospitals and are associated with healthcare-associated MRSA strains.

We aimed to evaluate the effects of sub-MICs of antibiotics on biofilm formation in clinical sequence type 5 (ST5) and ST72 MRSA isolates. ST5 and ST72 are the predominant MRSA clones with healthcare- and community-associated infections in South Korea, respectively (5–7). This study used three antibiotics (vancomycin, ciprofloxacin, and rifampin) commonly used to treat biofilm-related MRSA infections. We also include nafcillin for comparisons to three antibiotics because the effect of sub-MICs of β-lactam antibiotics on staphylococcal biofilm induction was well described in previous works (2–4, 8).

## MATERIALS AND METHODS

### Bacterial strains and antimicrobial agents

A total of 107 non-duplicate MRSA isolates were selected from patients with bloodstream infections between 2010 and 2016 at Gyeongsang National University Hospital (9). Nafcillin, vancomycin, ciprofloxacin, and rifampin were purchased from Sigma-Aldrich (St. Louis, MO, USA).

### Phenotypical and genotypical tests of MRSA isolates

Isolates were identified using the Vitek-2 system (bioMerieux, Marcy l’Etoile, France). The MICs of vancomycin, nafcillin, ciprofloxacin, and rifampin were determined using the broth microdilution method, according to the Clinical and Laboratory Standards Institute (CLSI) guidelines (10). CLSI susceptibility breakpoints were used to determine the susceptibility of MRSA isolates to nafcillin (≤2 mg/L), vancomycin (≤2 mg/L), ciprofloxacin (≤1 mg/L), and rifampin (≤1 mg/L) (11). All non-susceptible isolates were considered resistant. Multilocus sequence typing and staphylococcal protein A (*spa*) typing were performed according to previously described methods (12, 13). As described previously, the accessory gene regulator (*agr*) function was determined by the δ-hemolysin activity (14).

### Biofilm formation assays

Biofilm formation assays were conducted as described previously, with minor modifications (15). All cultures were incubated at 37°C under aerobic conditions. For each MRSA strain, the frozen stock was transferred to a sterile culture tube containing 3 mL of 0.5× tryptic soy broth (TSB) without glucose (Becton Dickinson and Co.), and the culture was grown overnight to stationary phase in a rotatory shaker. Bacterial cells were diluted to 5 × 10^5^ CFU/mL in 0.5× TSB (containing 1% glucose) supplemented with antibiotics at the indicated concentrations. Aliquots of cells (200 µL each) were transferred to the wells of a 96-well microtiter plate (catalog no. 3595, Corning), and the plate was incubated for 18 h. To quantify the extent of biofilm induction at the sub-MICs of antibiotics, MRSA isolates were cultured with vancomycin, nafcillin, ciprofloxacin, and rifampin at concentrations of 1/256×, 1/128×, 1/64×, 1/32×, 1/16×, 1/8×, 1/4×, and 1/2× MIC. Because the sub-MICs of rifampin were much lower than the clinically relevant concentrations of rifampin, we further tested the extent of biofilm induction at clinically relevant concentrations of rifampin (0.03, 0.06, 0.125, 0.25, 0.5, 1.0, 2.0, 4.0, 8.0, 16.0, and 32.0 mg/L) (16). To remove non-adherent planktonic bacteria, the plates were gently washed thrice with PBS. The remaining adherent bacteria (biofilms) were fixed at 60°C for at least 120 min and stained with a 0.1% crystal violet solution for 5 min. After washing the plates with deionized water, crystal violet-stained biofilms were solubilized in 33% glacial acetic acid for 1 h. The amount of crystal violet in each well was quantified by measuring optical density at 600 nm (OD_600_) using a spectrophotometer (Spark, TEKAN).

Positive [*S. aureus* American Type Culture Collection (ATCC) 29213, strong biofilm producer] and negative controls (*S. aureus* ATCC 25923, poor biofilm producer) were used to validate the variability between plates. All biofilm assays were performed in triplicate. The average coefficients of variation in OD_600_ for *S. aureus* ATCC 29213 and *S. aureus* ATCC 25923 were 7.1% and 5.3%, respectively.

### Interpretation of biofilm-forming ability and biofilm inducibility

The amount of biofilm formation (BF) was quantified by measuring the OD_600_ of the tested strains. It was further classified as follows: BF <cut-off OD (ODc) = no biofilm producer, BF <2 × ODc = weak biofilm producer, 2 × ODc < BF <4 × ODc = moderate biofilm producer, and BF >4 × ODc = strong biofilm producer (17). ODc for biofilm formation was defined as three standard deviations above the mean absorbance of the negative control (ATCC 25923). The cut-off OD values for biofilm increase (induction) or decrease in sub-MICs of antibiotics were calculated for each strain as three standard deviations above or below the mean OD of the tested strains in the absence of antibiotics (9). For statistical analyses, we categorized the cases of biofilm induction into weak and strong using a cut-off value of 1.5-fold induction.

### Field-emission scanning electron microscope analysis

The biofilm formation of MRSA at sub-MICs of antibiotics was visualized under field-emission scanning electron microscope (FESEM), as previously described with some modification (18). Bacterial suspensions containing sub-MICs of antibiotics and control suspensions (containing no antibiotics) were prepared in 24-well plates pre-placed with sterile cover glass slips. They were incubated at 37°C for 24 h and fixed with 2.5% glutaraldehyde overnight at 4°C. Biofilms underwent three 15-min washes with 0.1-M sodium cacodylate buffer to remove the fixative and were treated with 1% osmium tetraoxide for 1.5 h. After two washes with the buffer and one with tertiary distilled water, the samples were dehydrated using increasing concentrations of ethanol (30%, 50%, 70%, and 80%) for 15 min each. Biofilms were incubated with 95% ethanol for 30 min and 100% ethanol twice for 30 min each. Samples were cross-linked with a 1:1 mixture of hexamethyldisilizane (HMDS) and 100% ethanol for 15 min, followed by two rounds of HMDS alone for 15 min each. After removing HMDS, samples were dried overnight in a glass desiccator containing silica gel. Coverslips were mounted on stubs and coated with gold particles using an E-1045 ION sputter coater. Images were captured with a FESEM (S-4800; Hitachi, Tokyo, Japan).

### Statistical analyses

Categorical variables were compared using the chi-square test or Fisher’s exact test, as appropriate. The relationship between the basal level of biofilm formation and biofilm inducibility at sub-MICs of antibiotics was assessed using Pearson’s correlation coefficient. All tests for statistical significance were two tailed, and *P* values of ≤0.05 were considered statistically significant. All analyses were performed using R statistical software (version 4.2.3; R Foundation for Statistical Computing, Vienna, Austria).

## RESULTS

### Bacterial characteristics of MRSA isolates

The characteristics of the 107 clinical MRSA isolates are listed in Table 1. Of the 107 MRSA isolates tested, 63 (58.9%) and 44 (41.1%) belonged to the ST5 and ST72 lineages, respectively. ST5 MRSA represents clonal complex 5, typically referred to as the HA-MRSA strain. ST72 MRSA represents CC8, typically referred to as the CA-MRSA strain. All 107 isolates exhibited the inherent ability to produce biofilms in culture media without antibiotics: 19 (17.8%), 46 (43.0%), and 42 (39.2%) were weak, moderate, and strong biofilm producers, respectively. The ST72 isolates exhibited a stronger basal biofilm-forming ability than the ST5 isolates (77.3% vs 12.7%, *P* < 0.001). The *in vitro* antimicrobial susceptibilities of 107 clinical MRSA isolates are shown in Table 2.

**TABLE 1:** Bacterial characteristics of 107 clinical methicillin-resistant *Staphylococcus aureus* isolates^*b*^

Variable	HA-MRSA ST5 clone (*n* = 63)	CA-MRSA ST72 clone (*n* = 44)	*P* value
Source of MRSA bacteremia			
Intravascular catheters	32 (50.8)	5 (11.4)	<0.001
Bone and joint infection	3 (4.8)	9 (20.5)	0.03
Pneumonia	6 (9.5)	7 (15.9)	0.32
Skin and soft tissue infection	0	6 (13.6)	0.004
Surgical site infection	2 (3.2)	2 (4.5)	>0.99
Infective endocarditis	0	3 (6.8)	0.07
Other	4 (6.3)	5 (11.4)	0.48
Unknown	16 (25.4)	7 (15.9)	0.24
*spa* type			
t2460	35 (55.6)	0	<0.001
t002	15 (23.8)	0	<0.001
t9353	6 (9.5)	0	0.04
t664	0	23 (52.3)	<0.001
t324	0	13 (29.5)	<0.001
Other	7 (11.1)	8 (18.2)	0.30
Dysfunctional *agr*	46 (73.0)	4 (9.1)	<0.001
Basal biofilm-forming ability^*a*^			
Weak	18 (28.6)	1 (2.3)	<0.001
Moderate	37 (58.7)	9 (21.4)	<0.001
Strong	8 (12.7)	34 (77.3)	<0.001

^*a*^ Biofilm-forming ability in culture media without antibiotics.

^*b*^ *agr*, accessory gene regulator; CA-MRSA, community-associated methicillin-resistant *Staphylococcus aureus*; HA-MRSA, healthcare-associated methicillin-resistant *Staphylococcus aureus*; ST, sequence type.

**TABLE 2:** *In vitro* antimicrobial susceptibility of 107 clinical methicillin-resistant *Staphylococcus aureus* isolates^*c*^

Variable	All isolates (*n* = 107)	HA-MRSA ST5 clone (*n* = 63)	CA-MRSA ST72 clone (*n* = 44)
Nafcillin			
MIC_50_ (mg/L)	256	512	16
MIC_90_ (mg/L)	512	1,024	32
Range (mg/L)	4 to >1,024	4 to >1,024	8 to >256
Resistance, % (*n*/*N*)	100 (107/107)	100 (63/63)	100 (44/44)
Vancomycin			
MIC_50_ (mg/L)	1	1	1
MIC_90_ (mg/L)	2	2	1
Range (mg/L)	0.25–2.0	0.5–2.0	0.25–2.0
Resistance, % (*n*/*N*)	0 (0/107)	0 (0/63)	0 (0/44)
Ciprofloxacin			
MIC_50_ (mg/L)	64^*a*^	128	0.5
MIC_90_, mg/L	512^*a*^	512	2
Range (mg/L)	0.25–512.0	0.5–512.0	0.25–128.0
Resistance, % (*n*/*N*)	64 (69/107)	97 (61/63)	18 (8/44)
Rifampin			
MIC_50_ (mg/L)	0.008^*b*^	0.016	0.008
MIC_90_ (mg/L)	0.03^*b*^	0.5	0.016
Range (mg/L)	0.002–1,024	0.002–1,024	0.004–4.0
Resistance, % (*n*/*N*)	6 (7/107)	10 (6/63)	2 (2/44)

^*a*^ The MIC_50_/MIC_90_ values for the ciprofloxacin-susceptible (*n* = 38) and ciprofloxacin-resistant (*n* = 69) isolates were 0.5/2.0 and 128.0/512.0 mg/L, respectively.

^*b*^ The MIC_50_/MIC_90_ values for rifampin-susceptible (*n* = 100) and rifampin-resistant isolates (*n* = 7) were 0.008 and 0.016 mg/L, and 128/1024 mg/L, respectively.

^*c*^ MIC, minimum inhibitory concentration; NA, not available.

### Effect of sub-inhibitory concentrations of antibiotics on biofilm formation

**Fig 1:**
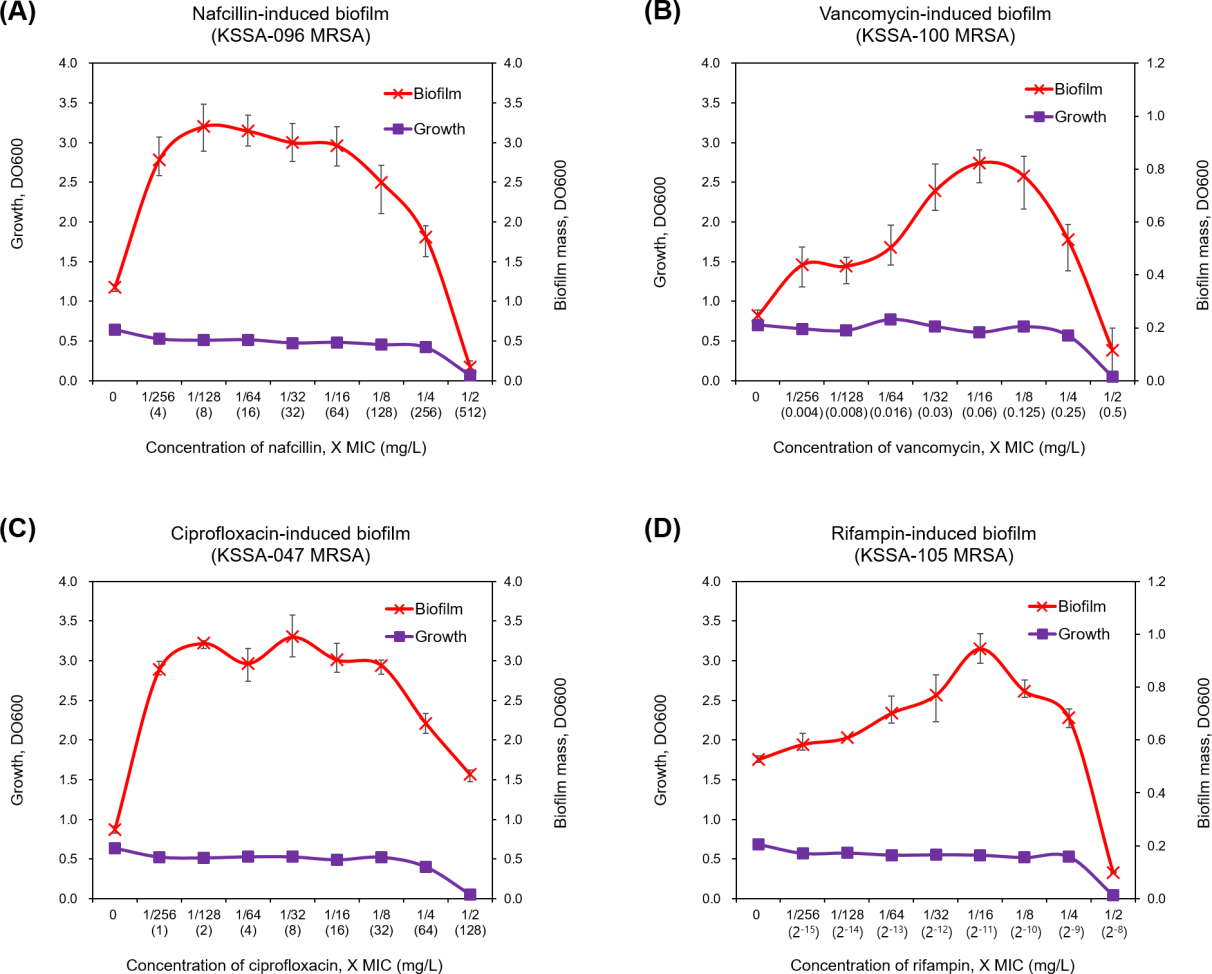
Representative patterns of dose-response curves for methicillin-resistant *S. aureus* biofilm induction at the sub-MICs of nafcillin (**A**), vancomycin (**B**), ciprofloxacin (**C**), and rifampin (**D**). The graphs show mean absorbance values from triplciated wells, and the error bars indicate standard deviations.

**Fig 2:**
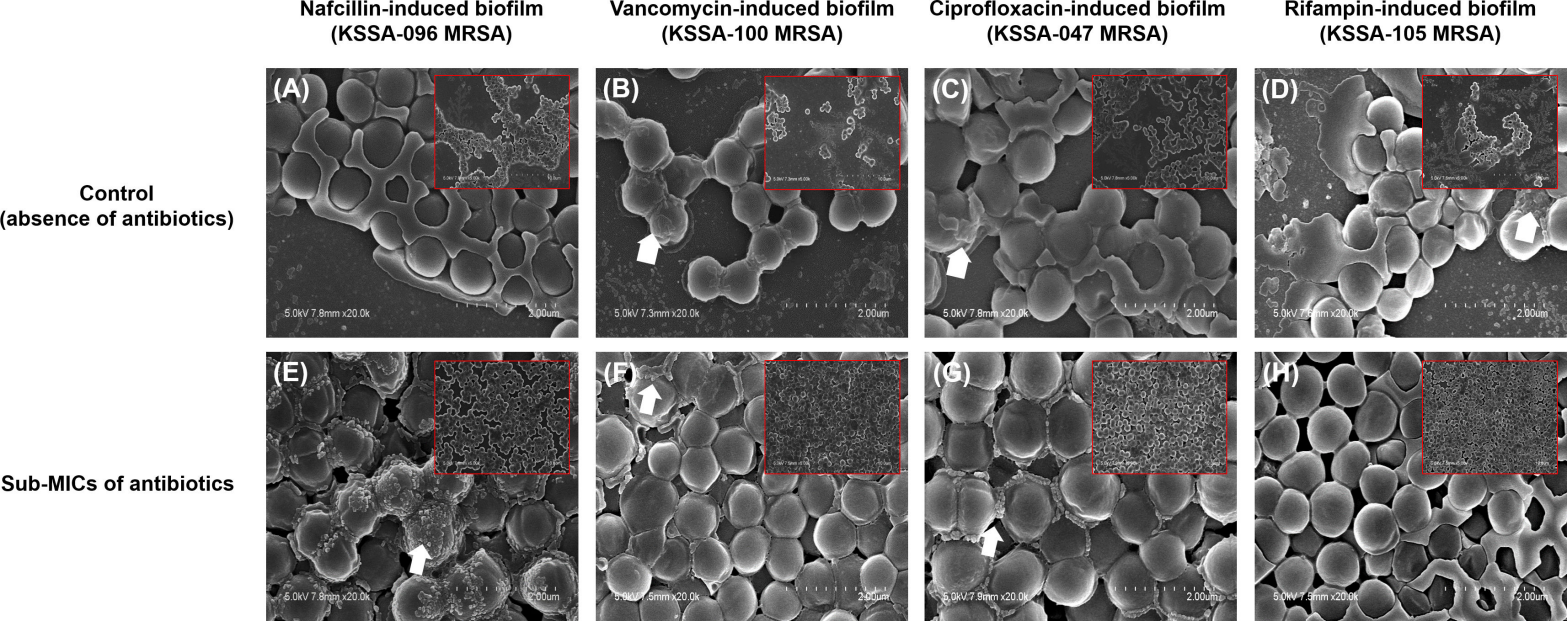
Scanning electron microscopy micrographs of the biofilm induction by the sub-inhibitory concentrations (sub-MICs) of 1/64 × MIC of nafcillin (**E**), 1/8 × MIC of vancomycin (**F**), 1/16 × MIC of ciprofloxacin (**G**), and 1/16 × MIC of rifampin (**H**). (A–D) Biofilm formation in cultures without antibiotics (controls). Inlet images indicate that MRSA biofilms treated with sub-MICs of antibiotics exhibited denser viable cells than untreated MRSA biofilms (×5,000 magnification). The main images indicate that sub-MICs of antibiotics did not distort cell morphology (×20,000 magnification). White arrows indicate the residual of the biofilm extracellular matrix.

**Fig 3:**
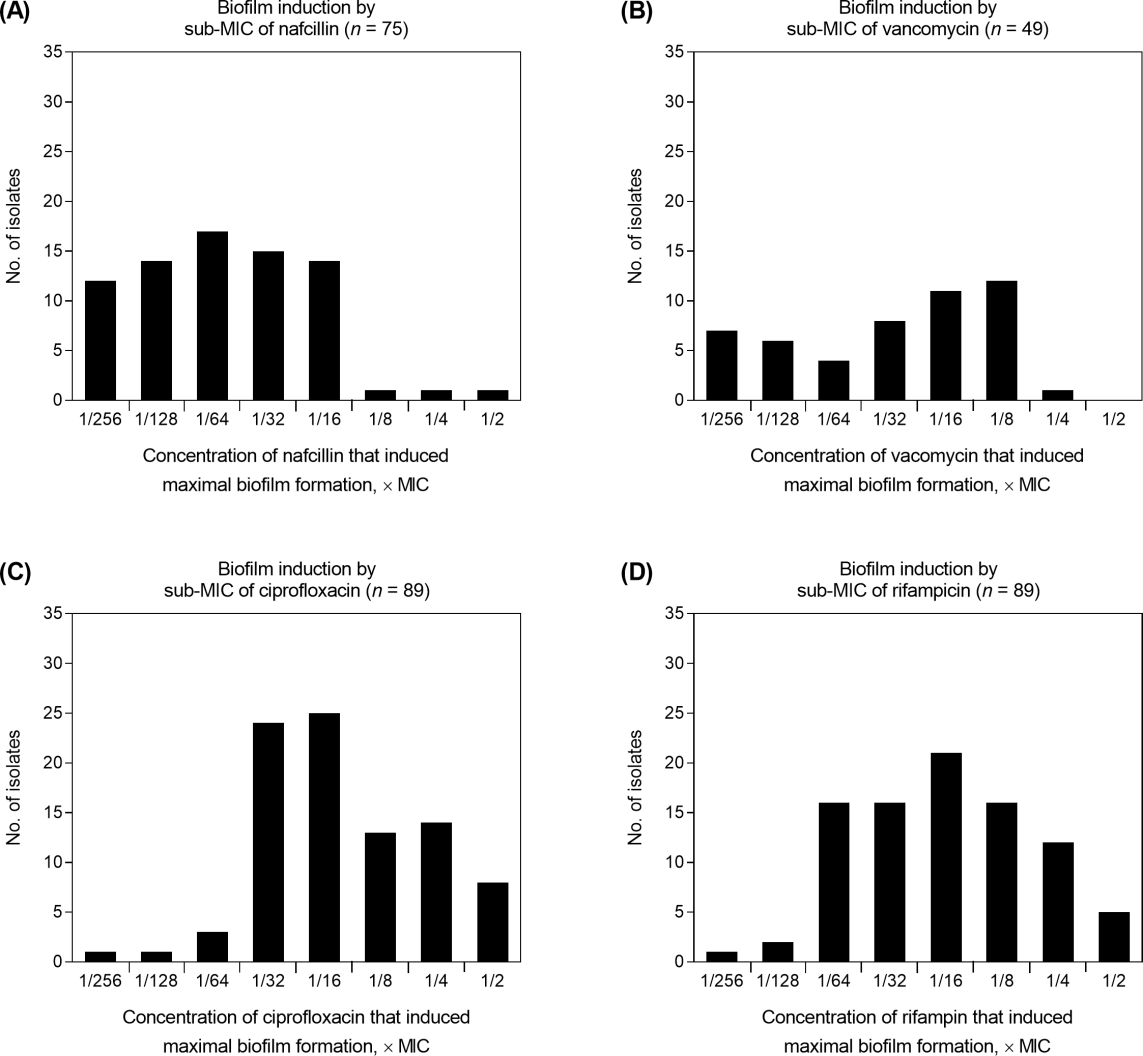
Distribution of the sub-inhibitory concentrations of nafcillin (**A**), vancomycin (**B**), ciprofloxacin (**C**), and rifampin (**D**) that induced maximal biofilm formation.

Figure 1 shows the representative patterns of dose-response curves for biofilm biomass induction by the sub-MICs of antibiotics. The growth patterns of planktonic bacteria within sub-MICs of antimicrobials were flat in all MRSA isolates (depicted by the purple line in Fig. 1). In contrast, the growth patterns of biofilm bacteria varied, depending on the strains. The most common pattern of dose-response curves for biofilm induction by sub-MICs of antibiotics is biphasic, characterized by low-dose stimulation of biofilm formation and high-dose inhibition (depicted by the red line in Fig. 1). We conducted scanning electron microscopy experiments to validate the biofilm induction at sub-MICs of antibiotics. The findings indicate that MRSA biofilms treated with sub-MICs of antibiotics exhibited denser viable cells than untreated MRSA biofilms. Sub-MICs of antibiotics did not distort cell morphology (Fig. 2). When MRSA isolates were cultured with a wide range of sub-MICs (1/256–1/2× MICs) of nafcillin, vancomycin, ciprofloxacin, and rifampin, biofilm formation was observed in 75 (70.1%), 49 (45.8%), 89 (83.2%), and 89 (83.2%) isolates, respectively. Among these isolates, peak biofilm induction occurred most frequently at 1/64×, 1/8×, 1/16×, and 1/16× the MICs of nafcillin, vancomycin, ciprofloxacin, and rifampin, respectively (Fig. 3). The effects of different sub-MICs of antibiotics on biofilm formation by MRSA isolates are shown in Table 3.

**TABLE 3:** Effects of different sub-inhibitory antimicrobial concentrations on biofilm formation in methicillin-resistant *Staphylococcus aureus* isolates

Concentration of antimicrobials		% (no. of isolates/total no. of isolates)		
×MIC^*a*^	Median (mg/L)	Increase	No effect	Decrease
Nafcillin				
1/2×	128	5 (5/107)	21 (22/107)	75 (80/107)
1/4×	64	19 (20/107)	24 (26/107)	57 (61/107)
1/8×	32	29 (31/107)	21 (22/107)	50 (54/107)
1/16×	16	37 (40/107)	26 (28/107)	36 (39/107)
1/32×	8	49 (52/107)	30 (32/107)	21 (23/107)
1/64×	4	50 (53/107)	38 (41/107)	12 (13/107)
1/128×	2	55 (59/107)	36 (39/107)	9 (9/107)
1/256×	1	51 (55/107)	42 (45/107)	7 (7/107)
Vancomycin				
1/2×	0.5	1 (1/107)	4 (4/107)	95 (102/107)
1/4×	0.25	9 (10/107)	24 (26/107)	66 (71/107)
1/8×	0.125	32 (34/107)	48 (51/107)	21 (22/107)
1/16×	0.06	28 (30/107)	65 (70/107)	7 (7/107)
1/32×	0.03	25 (27/107)	70 (75/107)	5 (5/107)
1/64×	0.016	17 (18/107)	76 (81/107)	7 (8/107)
1/128×	0.008	24 (26/107)	72 (77/107)	4 (4/107)
1/256×	0.004	28 (30/107)	71 (76/107)	1 (1/107)
Ciprofloxacin				
1/2×	32	9 (10/107)	6 (6/107)	85 (91/107)
1/4×	16	30 (32/107)	28 (30/107)	42 (45/107)
1/8×	8	51 (55/107)	34 (36/107)	15 (16/107)
1/16×	4	61 (65/107)	36 (39/107)	3 (3/107)
1/32×	2	66 (71/107)	32 (34/107)	2 (2/107)
1/64×	1	58 (62/107)	41 (44/107)	1 (1/107)
1/128×	0.5	45 (48/107)	54 (58/107)	1 (1/107)
1/256×	0.25	34 (36/107)	65 (70/107)	1 (1/107)
Rifampin				
1/2×	0.004	11 (12/107)	16 (17/107)	73 (78/107)
1/4×	0.002	45 (48/107)	23 (25/107)	32 (34/107)
1/8×	0.001	54 (58/107)	33 (35/107)	13 (14/107)
1/16×	0.0005	67 (72/107)	28 (30/107)	5 (5/107)
1/32×	0.00025	72 (77/107)	26 (28/107)	2 (2/107)
1/64×	0.000125	68 (73/107)	28 (30/107)	4 (4/107)
1/128×	0.000006	59 (63/107)	38 (41/107)	3 (5/107)
1/256×	0.000003	48 (51/107)	49 (52/107)	4 (4/107)

^*a*^ MIC, minimum inhibitory concentration.

### Microbiological factors associated with antibiotic-induced biofilm formations

To clarify the microbiological factors associated with biofilm induction by the sub-MICs of the antibiotics, we compared isolates exhibiting no biofilm induction with those with strong biofilm induction (Table 4). We assessed the extent of biofilm induction in culture media with antibiotic concentrations at which the strongest biofilm induction occurred most frequently (1/64× MICs for nafcillin, 1/8× MICs for vancomycin, and 1/16× MICs for ciprofloxacin and rifampin; Fig. 3). When MRSA isolates were cultured with 1/64× MICs of nafcillin, the ST5 strain and *agr* dysfunction were associated with strong biofilm induction, while the *spa* type t664 strain was associated with no biofilm induction. When the MRSA isolates were cultured with 1/8× MIC of the vancomycin, no microbiological factors were associated with strong biofilm induction. When MRSA isolates were cultured with 1/16× of the MICs of ciprofloxacin, the ST5 strain, *spa* type t002 strain, and ciprofloxacin resistance were associated with strong biofilm induction, whereas *spa* type t664 strain was associated with no biofilm induction. When MRSA isolates were cultured with 1/16× of the MICs of rifampin, ST5 was associated with strong biofilm induction.

**TABLE 4:** Microbiological factors associated with the capability of biofilm induction by sub-MICs of nafcillin, vancomycin, ciprofloxacin, and rifampin^*a*^

Variable	Nafcillin (1/64 × MIC)			Vancomycin (1/8 × MIC)			Ciprofloxacin (1/16 × MIC)			Rifampin (1/16 × MIC)		
	No biofilm induction (*n* = 54)	Strong Biofilm induction (*n* = 35)	*P* value	No biofilm induction (*n* = 73)	Strong biofilm induction (*n* = 13)	*P* value	No biofilm induction (*n* = 43)	Strong biofilm induction (*n* = 20)	*P* value	No biofilm induction (*n* = 35)	Strong biofilm induction (*n* = 36)	*P* value
MLST												
ST5	26 (48)	27 (77)	0.001	45 (62)	9 (69)	0.76	16 (37)	16 (80)	0.002	14 (40)	26 (72)	0.006
ST72	28 (52)	8 (23)		28 (38)	4 (31)		27 (63)	4 (20)		21 (60)	10 (28)	
*Spa* type												
t2460	14 (26)	16 (46)	0.053	25 (34)	5 (39)	0.76	14 (33)	4 (20)	0.30	8 (23)	14 (39)	0.14
t002	9 (17)	4 (11)	0.49	12 (16)	0	0.20	1 (2)	8 (40)	<0.001	6 (9)	5 (7)	0.70
t9353	1 (2)	3 (9)	0.30	4 (6)	2 (15)	0.22	0	1 (5)	0.32	0	3 (8)	0.24
t664	18 (33)	3 (9)	0.007	13 (18)	2 (15)	>0.99	13 (30)	1 (5)	0.03	11 (31)	6 (17)	0.15
t324	9 (17)	2 (6)	0.19	10 (14)	1 (8)	>0.99	8 (19)	3 (15)	>0.99	7 (20)	3 (8)	0.19
*agr* dysfunction	19 (35)	23 (66)	0.005	36 (49)	9 (69)	0.19	16 (37)	9 (45)	0.56	14 (40)	21 (58)	0.12
Antibiotic resistance	NA	NA	NA	NA	NA	NA	18 (42)	19 (95)	<0.001	3 (9)	1 (3)	0.36

^*a*^ *agr*, accessory gene regulator; MIC, minimum inhibitory concentration; MLST, multilocus sequence type; NA, not applicable ST, sequence type.

**Fig 4:**
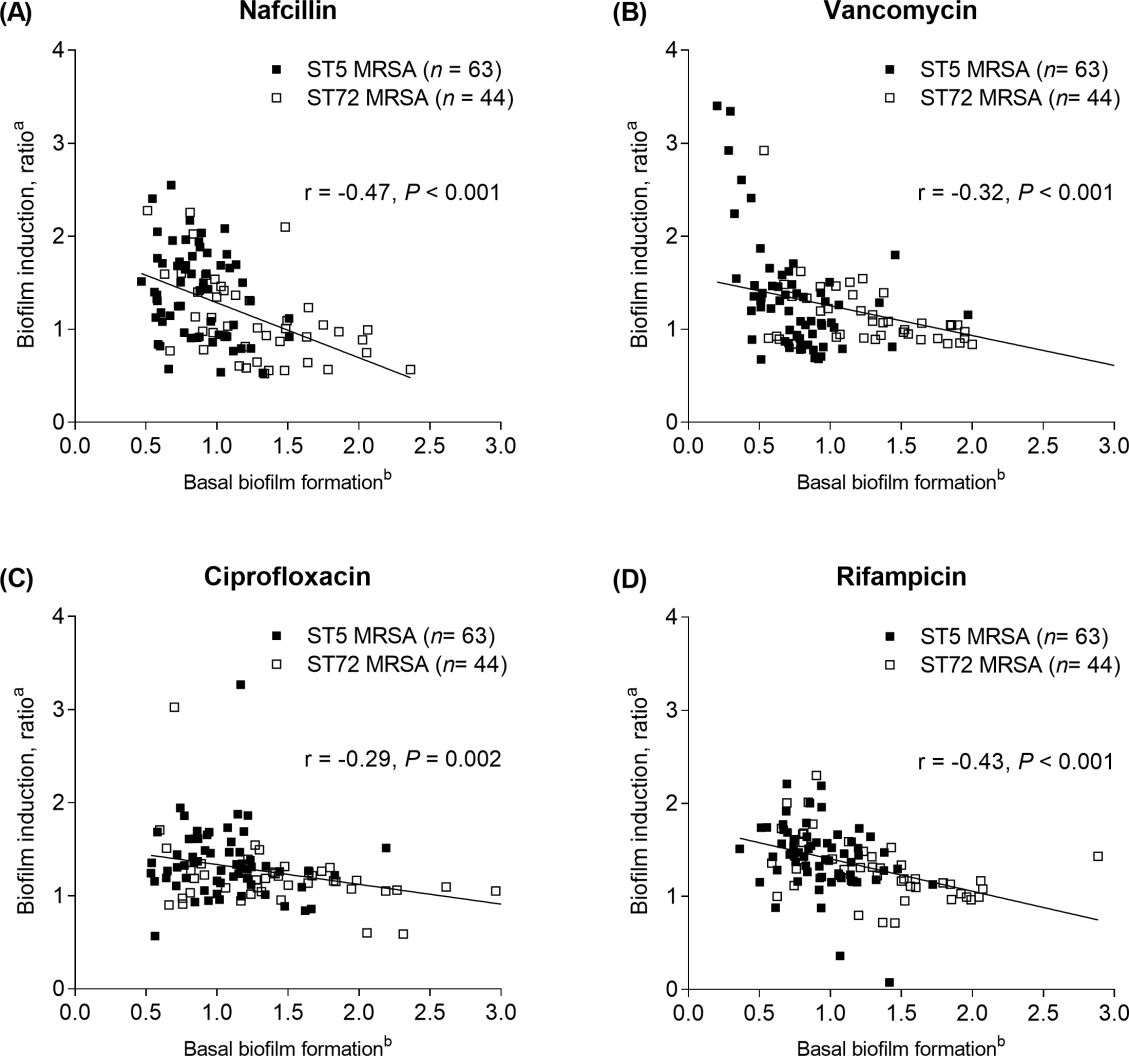
Relationship between basal level of biofilm formation and biofilm inducibility by 1/64 × MIC of nafcillin (**A**), 1/8 × MIC of vancomycin (**B**), 1/16 × MIC of ciprofloxacin (**C**), and 1/16 × MIC of rifampin (**D**). ^a^The relative antibiotic-induced biofilm formation compared with no antibiotics. ^b^The relative amount of basal biofilm formation was normalized to the amount of biofilm formation of ATCC 29213 in the absence of antibiotics, which is given a value of 1.

**Fig 5:**
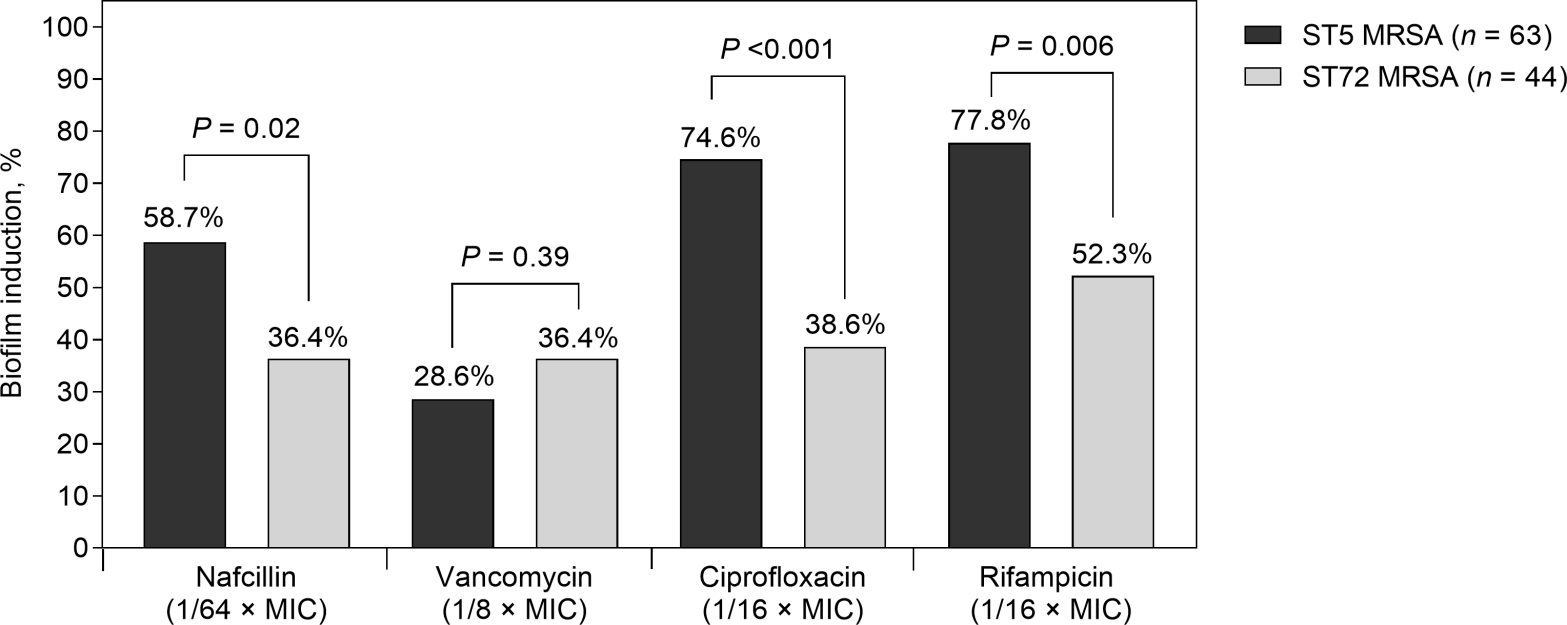
Comparison of the degree of biofilm induction between HA-MRSA (ST5) and CA-MRSA (ST72) in response to sub-inhibitory concentrations of nafcillin, vancomycin, ciprofloxacin, and rifampin.

The extent of biofilm production in the absence of antibiotics and biofilm induction at sub-MICs of nafcillin, vancomycin, ciprofloxacin, and rifampin were inversely correlated (Fig. 4). Figure 5 shows biofilm induction between ST5 MRSA and ST72 MRSA clones. Biofilm induction was more common in ST5 isolates than in ST72 isolates when the MRSA isolates were cultured with 1/64× MICs of nafcillin (58.7% vs 36.4%, *P* = 0.02), 1/16 × MICs of ciprofloxacin (74.6% vs 38.6%; *P* < 0.001), or 1/16 × MICs of rifampin (77.8% vs 52.3%, *P* = 0.006). This association was not evident when the MRSA isolates were cultured with 1/8× MIC of vancomycin (28.6% vs 36.4%, *P* = 0.39).

### Effect of clinically relevant concentrations of rifampin on biofilm formation

The sub-MICs of rifampin were much lower than the clinically relevant concentrations owing the low MICs of rifampin (MIC_50/90_ of 0.008/0.03 mg/L). Therefore, we tested the ability of rifampin to induce biofilm formation at 0.03-32 mg/L of rifampin. At these concentrations, rifampin-induced biofilm formation was rare in rifampin-susceptible MRSA [1.0% (1 of 100)] but common in rifampin-resistant MRSA [71.4% (5 of 7), *P* < 0.001]. The effects of different rifampin concentrations on biofilm formation by the MRSA isolates are summarized in Table 5.

**TABLE 5:** Effects of different rifampin concentrations on biofilm formation by the methicillin-resistant *Staphylococcus aureus* isolates

Concentration of rifampin (mg/L)	% (no. of isolates/total no. of isolates)		
	Increase	No effect	Decrease
Among rifampin-susceptible isolates			
32	0 (0/100)	0 (0/100)	100 (100/100)
16	0 (0/100)	0 (0/100)	100 (100/100)
8	0 (0/100)	0 (0/100)	100 (100/100)
4	0 (0/100)	0 (0/100)	100 (100/100)
2	0 (0/100)	0 (0/100)	100 (100/100)
1	0 (0/100)	0 (0/100)	100 (100/100)
0.5	0 (0/100)	0 (0/100)	100 (100/100)
0.25	0 (0/100)	0 (0/100)	100 (100/100)
0.125	0 (0/100)	0 (0/100)	100 (100/100)
0.06	0 (0/100)	0 (0/100)	100 (100/100)
0.03	1 (1/100)	1 (1/100)	98 (98/100)
Among rifampin-resistant isolates			
32	57 (4/7)	0 (0/7)	43 (3/7)
16	57 (4/7)	0 (0/7)	43 (3/7)
8	57 (4/7)	0 (0/7)	43 (3/7)
4	57 (4/7)	0 (0/7)	43 (3/7)
2	57 (4/7)	14 (1/7)	29 (2/7)
1	57 (4/7)	14 (1/7)	29 (2/7)
0.5	71 (5/7)	0 (0/7)	29 (2/7)
0.25	42 (3/7)	29 (2/7)	29 (2/7)
0.125	29 (2/7)	57 (4/7)	14 (1/7)
0.06	43 (3/7)	43 (3/7)	14 (1/7)
0.03	57 (4/7)	14 (1/7)	29 (2/7)

## DISCUSSION

In this study, we evaluated biofilm induction using wide range (1/256–1/2× MICs) of sub-MICs of four different classes of antibiotics in 107 clinical MRSA isolates. We found that the sub-MICs of nafcillin, vancomycin, ciprofloxacin, and rifampin frequently promoted biofilm formation in clinical MRSA isolates. The antibiotic concentrations that induced the maximum biofilm formation varied from 1/8× to 1/64× MICs, according to the antibiotic class. Most studies tested the effects of a single concentration of 1/4× or 1/2× MICs of antibiotics on bacterial biofilm formation (19). Our data suggest that testing biofilm formation at only 1/4× or 1/2× MICs of antibiotics may underestimate the biofilm induction ability in response to sub-MICs of antibiotics.

We evaluated the clinical and microbiological factors associated with antibiotic-induced biofilm formation in clinical MRSA isolates. HA-MRSA (ST5) showed a stronger ability to induce biofilm formation upon exposure to sub-MICs of nafcillin, ciprofloxacin, and rifampin than the Korean CA-MRSA strain (ST72). In comparison to our findings, Kaplan et al. reported that sub-MICs of methicillin induced biofilm formation in CA-MRSA strains (including USA300, USA 400, and USA 500) but did not induce biofilm formation in HA-MRSA strains (including COL, 252, and N315) (4). This discrepancy may be related to the difference in basal biofilm ability between the CA-MRSA strains included in our study vs the study by Kaplan et al. Kaplan et al. reported that CA-MRSA strains are weaker biofilm producers than HA-MRSA strains in culture media without antibiotics. Notably, the Korean CA-MRSA (ST72) strain was a stronger biofilm producer than the HA-MRSA strain (ST5) (9, 20). Previous studies have observed inverse correlations between bacterial biofilm-forming ability in the absence of antibiotics and the capacity for antibiotic-induced biofilm formation (21, 22). We confirmed this relationship in 107 clinical MRSA isolates from four antibiotic classes. Interestingly, the proportion of MRSA isolates that demonstrated strong biofilm induction after exposure to sub-MICs of vancomycin was lower than that of the other antibiotics. Additionally, there was no difference in the biofilm formation ability between HA- and CA-MRSA strains at the sub-MICs of vancomycin. A previous study on representative MRSA strains also revealed that sub-MICs of vancomycin significantly promoted *sarA*-dependent biofilm formation in both CA- and HA-MRSA strains (23). Although we did not investigate the composition of the biofilm mass or the expression of biofilm-related genes, our experimental results suggest the possibility of a different mechanism of biofilm induction after exposure to sub-MICs of vancomycin compared with other antibiotics.

The *agr* quorum-sensing system modulated *S. aureus* biofilm formation and cell dispersal from biofilm (19). Previous studies reported that *agr* defective isolates or mutants exhibit a higher propensity to form *S. aureus* biofilms than *agr* functional isolates (24, 25). There is limited data on the relationship between *agr* dysfunction and antibiotic-induced biofilm formation. We found an association between *agr* dysfunction and biofilm induction upon exposure to the sub-MICs of nafcillin; however, this association was not evident with the sub-MICs of vancomycin, ciprofloxacin, and rifampin. In the present study, ciprofloxacin-induced biofilm formation was more common in ciprofloxacin-resistant strains than in ciprofloxacin-susceptible strains. This association was not observed in rifampin-induced biofilm formation. In a recent study, clindamycin-resistant MRSA strains exhibited more frequent exhibited biofilm induction upon clindamycin exposure than clindamycin-susceptible MRSA strains [33% (2 of 6) vs 100% (7 of 7), respectively] (26). Overall, our results suggest that the modulation of MRSA biofilm formation upon antibiotic exposure is a complex phenomenon and depends on several factors, such as bacterial strain, antibiotic class, antibiotic resistance, and *agr* dysfunction.

The benefits of rifampin for staphylococcal biofilm-related infections are well documented based on *in vitro*, animal, and clinical data (27). Our *in vitro* data suggested that sub-MICs of rifampin can induce biofilm formation in clinical MRSA strains. However, in clinical situations, rifampin-susceptible MRSA isolates are the least likely to be exposed to sub-MICs of rifampin because of its low MICs [MIC_50_ = 0.008 mg/L in the current study and a recent study (28)]. Therefore, we evaluated the effect of rifampin at clinically relevant concentrations of 0.03-32 mg/L. Rifampin at these concentrations rarely augmented biofilm formation in rifampin-susceptible MRSA isolates (1%) but frequently augmented biofilm formation in rifampin-resistant MRSA isolates (71%). Our results indicate that the indiscriminate use of empirical rifampin before obtaining susceptibility results should be discouraged. The emergence of rifampin resistance during the therapy is common in staphylococcal biofilm-related infections, including infective endocarditis (29, 30), periprosthetic joint infections (31), and osteomyelitis (32). In this case, continuing rifampin treatment may have augmented MRSA biofilm formation and led to treatment failure. Therefore, every effort should be made to prevent the emergence of rifampin resistance, including maintaining effective bactericidal concentrations of the companion antibiotic of rifampin (such as vancomycin) and adding rifampin to standard therapy after appropriate surgical treatment (33).

This study has several limitations. First, we evaluated the effects of antibiotic sub-MICs on the biofilm formation by CA-MRSA and HA-MRSA strains that are predominant in South Korea. Our findings cannot be generalized to other countries with different circulating HA-MRSA and CA-MRSA isolates. Second, the differences in biofilm induction between ST5 HA-MRSA and ST72 CA-MRSA may result from other strain-associated factors that we did not examine rather than the strain itself.

In conclusion, the sub-MICs of antibiotics frequently promote biofilm induction in clinical MRSA isolates. Antibiotic-induced biofilm formation depends on the antibiotic class, MRSA strain, and antibiotic resistance. Our results emphasize the importance of maintaining effective bactericidal concentrations of antibiotics to treat biofilm-related infections.

## AFTER EPUB

*[This article was published on 23 April 2024 with an error in the title. The title was corrected in the current version, posted on 29 April 2024.]*

## References

[1] Percival SL, Suleman L, Vuotto C, Donelli G. 2015. Healthcare-associated infections, medical devices and biofilms: risk, tolerance and control. J Med Microbiol 64:323–334. doi:10.1099/jmm.0.000032 25670813

[2] Ng M, Epstein SB, Callahan MT, Piotrowski BO, Simon GL, Roberts AD, Keiser JF, Kaplan JB. 2014. Induction of MRSA biofilm by low-dose beta-lactam antibiotics: specificity, prevalence and dose-response effects. Dose Response 12:152–161. doi:10.2203/dose-response.13-021.Kaplan 24659939 PMC3960960

[3] Mlynek KD, Callahan MT, Shimkevitch AV, Farmer JT, Endres JL, Marchand M, Bayles KW, Horswill AR, Kaplan JB. 2016. Effects of low-dose amoxicillin on Staphylococcus aureus USA300 biofilms. Antimicrob Agents Chemother 60:2639–2651. doi:10.1128/AAC.02070-15 26856828 PMC4862544

[4] Kaplan JB, Izano EA, Gopal P, Karwacki MT, Kim S, Bose JL, Bayles KW, Horswill AR. 2012. Low levels of beta-lactam antibiotics induce extracellular DNA release and biofilm formation in Staphylococcus aureus. mBio 3:e00198-12. doi:10.1128/mBio.00198-12 22851659 PMC3419523

[5] Park KH, Chong YP, Kim SH, Lee SO, Choi SH, Lee MS, Jeong JY, Woo JH, Kim YS. 2015. Community-associated MRSA strain ST72-SCCmecIV causing bloodstream infections: clinical outcomes and bacterial virulence factors. J Antimicrob Chemother 70:1185–1192. doi:10.1093/jac/dku475 25433004

[6] Choi SH, Lee J, Jung J, Kim ES, Kim MJ, Chong YP, Kim SH, Lee SO, Choi SH, Woo JH, Kim YS. 2021. A longitudinal study of adult patients with Staphylococcus aureus bacteremia over 11 years in Korea. J Korean Med Sci 36:e104. doi:10.3346/jkms.2021.36.e104 33904260 PMC8076844

[7] Park C, Lee DG, Kim SW, Choi SM, Park SH, Chun HS, Choi JH, Yoo JH, Shin WS, Kang JH, Kim JH, Lee SY, Kim SM, Pyun BY. 2007. Predominance of community-associated methicillin-resistant Staphylococcus aureus strains carrying staphylococcal chromosome cassette mec type IVA in South Korea. J Clin Microbiol 45:4021–4026. doi:10.1128/JCM.01147-07 17942660 PMC2168574

[8] Frank KL, Reichert EJ, Piper KE, Patel R. 2007. In vitro effects of antimicrobial agents on planktonic and biofilm forms of Staphylococcus lugdunensis clinical isolates. Antimicrob Agents Chemother 51:888–895. doi:10.1128/AAC.01052-06 17158933 PMC1803120

[9] Park KH, Jung M, Kim DY, Lee YM, Lee MS, Ryu BH, Hong SI, Hong KW, Bae IG, Cho OH. 2020. Effects of subinhibitory concentrations of chlorhexidine and mupirocin on biofilm formation in clinical meticillin-resistant Staphylococcus aureus. J Hosp Infect 106:295–302. doi:10.1016/j.jhin.2020.07.010 32679053

[10] Clinical and Laboratory Standards Institute. 2015. Methods for dilution antimicrobial susceptibility tests for bacteria that grow aerobically: approved standard. 10th ed. CLSI, Wayne, PA, USA.

[11] Clinical and Laboratory Standards Institute. 2020. Performance standards for antimicrobial susceptibility testing; approved standard. 30th ed. CLSI, Wayne, PA, USA.

[12] Enright MC, Day NP, Davies CE, Peacock SJ, Spratt BG. 2000. Multilocus sequence typing for characterization of methicillin-resistant and methicillin-susceptible clones of Staphylococcus aureus. J Clin Microbiol 38:1008–1015. doi:10.1128/JCM.38.3.1008-1015.2000 10698988 PMC86325

[13] Shopsin B, Gomez M, Montgomery SO, Smith DH, Waddington M, Dodge DE, Bost DA, Riehman M, Naidich S, Kreiswirth BN. 1999. Evaluation of protein A gene polymorphic region DNA sequencing for typing of Staphylococcus aureus strains. J Clin Microbiol 37:3556–3563. doi:10.1128/JCM.37.11.3556-3563.1999 10523551 PMC85690

[14] Traber KE, Lee E, Benson S, Corrigan R, Cantera M, Shopsin B, Novick RP. 2008. agr function in clinical Staphylococcus aureus isolates. Microbiology (Reading) 154:2265–2274. doi:10.1099/mic.0.2007/011874-0 18667559 PMC4904715

[15] Kwasny SM, Opperman TJ. 2010. Static biofilm cultures of Gram-positive pathogens grown in a microtiter format used for anti-biofilm drug discovery. Curr Protoc Pharmacol 13. doi:10.1002/0471141755.ph13a08s50 PMC3272335 22294365

[16] Prahl JB, Johansen IS, Cohen AS, Frimodt-Møller N, Andersen ÅB. 2014. Clinical significance of 2 h plasma concentrations of first-line anti-tuberculosis drugs: a prospective observational study. J Antimicrob Chemother 69:2841–2847. doi:10.1093/jac/dku210 25140577

[17] Stepanović S, Vuković D, Hola V, Di Bonaventura G, Djukić S, Cirković I, Ruzicka F. 2007. Quantification of biofilm in microtiter plates: overview of testing conditions and practical recommendations for assessment of biofilm production by staphylococci. APMIS 115:891–899. doi:10.1111/j.1600-0463.2007.apm_630.x 17696944

[18] Ibberson CB, Parlet CP, Kwiecinski J, Crosby HA, Meyerholz DK, Horswill AR. 2016. Hyaluronan modulation impacts Staphylococcus aureus biofilm infection. Infect Immun 84:1917–1929. doi:10.1128/IAI.01418-15 27068096 PMC4907140

[19] Kaplan JB. 2011. Antibiotic-induced biofilm formation. Int J Artif Organs 34:737–751. doi:10.5301/ijao.5000027 22094552

[20] Cha JO, Park YK, Lee YS, Chung GT. 2011. In vitro biofilm formation and bactericidal activities of methicillin-resistant Staphylococcus aureus clones prevalent in Korea. Diagn Microbiol Infect Dis 70:112–118. doi:10.1016/j.diagmicrobio.2010.11.018 21398072

[21] Kaplan JB, Jabbouri S, Sadovskaya I. 2011. Extracellular DNA-dependent biofilm formation by Staphylococcus epidermidis RP62A in response to subminimal inhibitory concentrations of antibiotics. Res Microbiol 162:535–541. doi:10.1016/j.resmic.2011.03.008 21402153 PMC3109171

[22] Pérez-Giraldo C, Rodríguez-Benito A, Morán FJ, Hurtado C, Blanco MT, Gómez-García AC. 1994. In-vitro slime production by Staphylococcus epidermidis in presence of subinhibitory concentrations of ciprofloxacin, ofloxacin and sparfloxacin. J Antimicrob Chemother 33:845–848. doi:10.1093/jac/33.4.845 8056703

[23] Abdelhady W, Bayer AS, Seidl K, Moormeier DE, Bayles KW, Cheung A, Yeaman MR, Xiong YQ. 2014. Impact of vancomycin on sarA-mediated biofilm formation: role in persistent endovascular infections due to methicillin-resistant Staphylococcus aureus. J Infect Dis 209:1231–1240. doi:10.1093/infdis/jiu007 24403556 PMC3969550

[24] Vuong C, Saenz HL, Götz F, Otto M. 2000. Impact of the agr quorum-sensing system on adherence to polystyrene in Staphylococcus aureus. J Infect Dis 182:1688–1693. doi:10.1086/317606 11069241

[25] Valour F, Rasigade J-P, Trouillet-Assant S, Gagnaire J, Bouaziz A, Karsenty J, Lacour C, Bes M, Lustig S, Bénet T, Chidiac C, Etienne J, Vandenesch F, Ferry T, Laurent F, Lyon BJI Study Group. 2015. Delta-toxin production deficiency in Staphylococcus aureus: a diagnostic marker of bone and joint infection chronicity linked with osteoblast invasion and biofilm formation. Clin Microbiol Infect 21:568. doi:10.1016/j.cmi.2015.01.026 25677632

[26] Schilcher K, Andreoni F, Dengler Haunreiter V, Seidl K, Hasse B, Zinkernagel AS. 2016. Modulation of Staphylococcus aureus biofilm matrix by subinhibitory concentrations of clindamycin. Antimicrob Agents Chemother 60:5957–5967. doi:10.1128/AAC.00463-16 27458233 PMC5038281

[27] Zimmerli W, Sendi P. 2019. Role of rifampin against staphylococcal biofilm infections in vitro, in animal models, and in orthopedic-device-related infections. Antimicrob Agents Chemother 63:e01746-18. doi:10.1128/AAC.01746-18 30455229 PMC6355554

[28] Albano M, Karau MJ, Greenwood-Quaintance KE, Osmon DR, Oravec CP, Berry DJ, Abdel MP, Patel R. 2019. In vitro activity of rifampin, rifabutin, rifapentine, and rifaximin against planktonic and biofilm states of staphylococci isolated from periprosthetic joint infection. Antimicrob Agents Chemother 63:e00959-19. doi:10.1128/AAC.00959-19 31451499 PMC6811393

[29] Levine DP, Fromm BS, Reddy BR. 1991. Slow response to vancomycin or vancomycin plus rifampin in methicillin-resistant Staphylococcus aureus endocarditis. Ann Intern Med 115:674–680. doi:10.7326/0003-4819-115-9-674 1929035

[30] Karchmer AW, Archer GL, Dismukes WE. 1983. Rifampin treatment of prosthetic valve endocarditis due to Staphylococcus epidermidis. Rev Infect Dis 5:S543–S548. doi:10.1093/clinids/5.supplement_3.s543 6556711

[31] Achermann Y, Eigenmann K, Ledergerber B, Derksen L, Rafeiner P, Clauss M, Nüesch R, Zellweger C, Vogt M, Zimmerli W. 2013. Factors associated with rifampin resistance in staphylococcal periprosthetic joint infections (PJI): a matched case-control study. Infection 41:431–437. doi:10.1007/s15010-012-0325-7 22987291

[32] Daver NG, Shelburne SA, Atmar RL, Giordano TP, Stager CE, Reitman CA, White AC. 2007. Oral step-down therapy is comparable to intravenous therapy for Staphylococcus aureus osteomyelitis. J Infect 54:539–544. doi:10.1016/j.jinf.2006.11.011 17198732

[33] Coiffier G, Albert JD, Arvieux C, Guggenbuhl P. 2013. Optimizing combination rifampin therapy for staphylococcal osteoarticular infections. Joint Bone Spine 80:11–17. doi:10.1016/j.jbspin.2012.09.008 23332140

